# Towards Detection of Glycoproteins Using Molecularly Imprinted Nanoparticles and Boronic Acid-Modified Fluorescent Probe

**DOI:** 10.3390/polym11010173

**Published:** 2019-01-18

**Authors:** Lingdong Jiang, Rui Lu, Lei Ye

**Affiliations:** 1 Division of Pure and Applied Biochemistry, Department of Chemistry, Lund University, Lund 22100 Sweden; lingdong.jiang@tbiokem.lth.se (L.J.); rlu@njust.edu.cn (R.L.); 2 Jiangsu Key Laboratory of Chemical Pollution Control and Resources Reuse, School of Environmental and Biological Engineering, Nanjing University of Science and Technology, Nanjing 210094, China

**Keywords:** molecular imprinting, glycoprotein, boronic acid, fluorescent probe

## Abstract

Glycoproteins represent a group of important biomarkers for cancer and other life-threatening diseases. Selective detection of specific glycoproteins is an important step for early diagnosis. Traditional glycoprotein assays are mostly based on lectins, antibodies, and enzymes, biochemical reagents that are costly and require special cold chain storage and distribution. To address the shortcomings of the existing glycoprotein assays, we propose a new approach using protein-imprinted nanoparticles to replace the traditional lectins and antibodies. Protein-imprinted binding sites were created on the surface of silica nanoparticles by copolymerization of dopamine and aminophenylboronic acid. The imprinted nanoparticles were systematically characterized by dynamic light scattering, scanning and transmission electron microscopy, thermogravimetric analysis, Fourier transform infrared spectroscopy, and elemental analysis. A boronic acid-modified fluorescent probe was used to detect the target glycoprotein captured by the imprinted nanoparticles. Using horseradish peroxidase as a model glycoprotein, we demonstrated that the proposed method can be applied to detect target protein containing multiple glycosylation sites. Because of their outstanding stability and low cost, imprinted nanoparticles and synthetic probes are attractive replacements of traditional biochemical reagents to develop simpler, faster, and more cost-effective analytical methods for glycoproteins.

## 1. Introduction

As one important group of disease biomarkers, glycoproteins are frequently measured in biological samples for early diagnosis [1]. The increased use of advanced mass spectrometry in glycoproteomics has brought in significant improvement in the practical analysis of glycoproteins [2]. Nevertheless, for routine monitoring and fast screening purposes, it is more attractive to perform glycoprotein assays without involving heavy analytical instruments under specialized laboratory conditions. Traditional glycoprotein assays are often based on lectins, antibodies, and enzymes to realize selective capture and detection/quantification of target glycoproteins [3]. As lectins, antibodies and enzymes are expensive and have limited shelf life; it is therefore desirable for synthetic substitutes of the biochemical reagents to be developed and used to achieve fast and simplified glycoprotein assays.

Molecular imprinting is a template-guided synthetic process to create specific binding sites in cross-linked polymer materials [4,5,6,7]. Over the past few years, this synthetic technique has been extended to generate specific binding sites for proteins and cells on a variety of material surfaces [8,9,10,11]. Molecularly imprinted polymers (MIPs) have significant advantages in terms of stability and reusability [12]. In the literature, several MIPs have been described to bind glycoproteins via selective boronic acid–glycan interactions [13,14,15,16,17,18]. Because the formation of a covalent bond between boronic acid and the *cis*–diol structure in the carbohydrate moiety of glycosylated proteins can be accomplished in an aqueous solution, MIPs designed for glycoprotein recognition are often prepared from boronic acid-based building blocks. To maintain an intact three-dimensional structure of the template proteins to create well-defined protein binding sites, it is often necessary to carry out protein imprinting in an aqueous solution at ambient temperature. The self-polymerization of dopamine has proven a very useful approach to preparing protein-imprinted thin polymer films [19,20,21,22]. Previous investigations on glycoprotein-imprinting have mainly focused on developing MIPs for selective protein binding. There were only a few publications related to MIP-based sandwich assay for proteins [23]. In one example, Liu and co-workers used a protein-imprinted polymer to capture glycoprotein, and boronic acid-coated silver nanoparticles as analytical probe to quantify the target protein by measuring the surface-enhanced Raman scattering signal of the boronic acid [24]. 

The aim of this work was to investigate the feasibility of detecting MIP-captured glycoproteins using boronic acid-conjugated fluorescent probe. We selected a sandwich format for protein identification based on the following hypothesis: As glycoproteins have different glycosylation patterns, imprinted binding site and boronic acid–fluorophore conjugate can be designed to recognize different epitopes on a single glycoprotein. In this way, an analytical readout may be made more target-specific depending on the fidelity of imprinting and the unique glycosylation pattern of the target protein. In this proof-of-principle work, we selected three glycoproteins with different glycosylation patterns as model templates to prepare surface-imprinted polydopamine on silica nanoparticles. The glycoproteins captured by the imprinted core–shell nanoparticles were detected by boronic acid–fluorescein conjugate. Among the three glycoproteins, i.e., horseradish peroxidase, ovalbumin, and transferrin, horseradish peroxidase was successfully identified using the protein-imprinted nanoparticles combined with fluorescence intensity measurement.

## 2. Materials and Methods

### 2.1. Materials

Tetraethylorthosilicate (TEOS, reagent grade, 98%), methanol (HPLC, ≥99.9%), ammonia, (3-aminopropyl)-triethoxysilane (APTES, ≥98%), toluene (anhydrous, 99.8%), triethylamine (TEA, ≥98%), acetone (HPLC, ≥99.9%), 4-formylphenylboronic acid (FPBA, ≥95%), sodium cyanoborohydride (NaBH_3_CN, reagent grade, 95%), dopamine hydrochloride, ammonium persulfate (APS, ≥98%), m-aminophenylboronic acid monohydrate (APBA, 98%), fluorescein isothiocyanate (FITC, ≥90%), bovine serum albumin (BSA, ≥98%), ovalbumin (OVA, ≥98%), transferrin (TRF, ≥98%), horseradish peroxidase (HRP, ~150 U mg^−1^), acetic acid (≥99%), and sodium dodecyl sulfate (SDS, ≥99%) were purchased from Sigma-Aldrich or Fisher (Steinheim, Germany) and were used as received. Ultrapure water (18.2 MΩ cm) was prepared by an ELGA LabWater System (Veolia, High Wycombe, UK).

### 2.2. Synthesis of Silica Nanoparticles

Silica nanoparticles were synthesized using a one-step Stöber method [25]. Briefly, a mixture of water (33 mL), methanol (100 mL), and ammonia (25%, 22.4 mL) was introduced into a round bottom flask. After addition of 130 mL methanol containing 13.8 mL TEOS, the solution was magnetically stirred at 20 °C for 12 h. After the reaction, the silica nanoparticles formed were separated by centrifugation to remove the unreacted ammonia and TEOS. The silica nanoparticles were washed thoroughly with water and methanol, and then dried in a vacuum oven.

### 2.3. Synthesis of Amino-Functionalized Silica Nanoparticles (Si@NH_2_)

Silica nanoparticles (1.5 g) were added to a round bottom flask, followed by the addition of 1% APTES solution (0.5 mL APTES dissolved in 49.5 mL anhydrous toluene). The suspension was magnetically stirred at reflux temperature for 24 h. The particles were isolated by centrifugation, washed with acetone (20 mL, 3 times) and methanol (20 mL, 3 times), and dried in a vacuum oven.

### 2.4. Synthesis of Boronic Acid–Functionalized Silica Nanoparticles (Si@BA)

Si@NH_2_ particles (1.2 g) were suspended in 30 mL of FPBA solution in methanol (1 mg mL^−1^). The suspension was magnetically stirred at 20 °C for 18 h. After the reaction, the particles were separated by centrifugation, washed with methanol (20 mL, 3 times), and then immersed in 30 mL of NaBH_3_CN solution in methanol (1 mg mL^−1^). The mixture was magnetically stirred at 20 °C for another 22 h. Finally, the particles were thoroughly washed with water and methanol, and then dried in a vacuum oven.

### 2.5. Preparation of Protein-Imprinted Core–Shell Nanoparticles 

Si@BA particles (3 mg) were suspended in 0.5 mL phosphate buffer (0.1 M, pH 8.5). The particle suspension was mixed with 0.5 mL solution of different glycoproteins (HRP, OVA, and TRF, 2 mg mL^−1^) and incubated for 8 h. The particles were collected by centrifugation and washed with the same buffer. Protein-imprinted thin polymer film was prepared on the nanoparticle surface using the self-polymerization of dopamine in alkaline buffer [26]. The protein-immobilized nanoparticles were transferred into 1 mL solution containing 1.6 mg APBA, 2.0 mg dopamine hydrochloride, and 1.2 mg APS freshly prepared in phosphate buffer (0.1 M, pH 8.5). The reaction mixture was gently stirred at room temperature for 14 h. After the reaction, the nanoparticles were collected by centrifugation, and washed thoroughly with 0.1 M acetic acid containing 10% SDS (*w*/*v*) to remove the protein template. BSA was used as a nonglycosylated protein template to prepare a reference material for comparison. Non-imprinted polymer (NIP) particles were prepared following the same procedure except that no protein template was immobilized on the Si@BA particles.

### 2.6. Synthesis of Boronic Acid–Fluorescein Conjugate (BA–FITC) 

Boronic acid-modified fluorescent dye was synthesized following a protocol described by Bao et al. [27] with minor modification. Briefly, APBA (15.6 mg, 0.1 mmol) and FITC (39.1 mg, 0.1 mmol) were dissolved in 25 mL methanol. The solution was stirred at 30 °C in dark for 24 h. After the reaction, the solvent was removed using a rotary evaporator. The crude product was purified by silica column chromatography using ethyl acetate:ethanol (5:1) as solvent. The final product was obtained as yellow-orange powder. 

### 2.7. Detection of Glycoprotein Captured by Imprinted Core–Shell Nanoparticles 

Protein-imprinted nanoparticles (3 mg) were added to 1 mL of protein solution (0.2−1.0 mg mL^−1^) prepared in phosphate buffer (0.1 M, pH 8.5). The suspension was incubated at 20 °C for 4 h. The nanoparticles were collected by centrifugation and washed with the same buffer to remove the unbound protein. After this step, the nanoparticles were transferred into 1 mL of BA–FITC solution (0.1 mM) prepared in phosphate buffer (0.1 M, pH 8.5). The mixture was incubated in dark for 14 h. After separating the particles by centrifugation, the amount of unbound BA–FITC in the supernatant was quantified by fluorescence intensity measurement (Ex: 492 nm, Em: 512 nm). 

### 2.8. Characterization 

Thermal gravimetric analysis (TGA) was carried out under a nitrogen atmosphere. The samples were heated to 900 °C at 10 °C min^−1^ and held for 30 min. Elemental analysis (C, H, N, B) was performed by Mikroanalytisches Laboratorium Kolbe (Oberhausen, Germany). UV-Vis absorption spectra were recorded using a Cary 60 UV/Vis spectrophotometer (Agilent Technologies, Mulgrave, Victoria, Australia). Fluorescence emission was measured using a QuantaMaster C-60/2000 spectrofluorometer (Photon Technology International, Lawrenceville, NJ, USA). The surface morphologies of particles were observed using a scanning electron microscope (SEM) (JSM-6700F, JEOL, Tokyo, Japan) and a transmission electron microscope (TEM) (JEM-1400 Plus). The hydrodynamic diameters of the nanoparticles were measured in water at 25 °C by dynamic light scattering (DLS) using a Zetasizer Nano ZS instrument (Malvern Instruments, Worcestershire, UK).

## 3. Results and Discussion

**Scheme 1:**
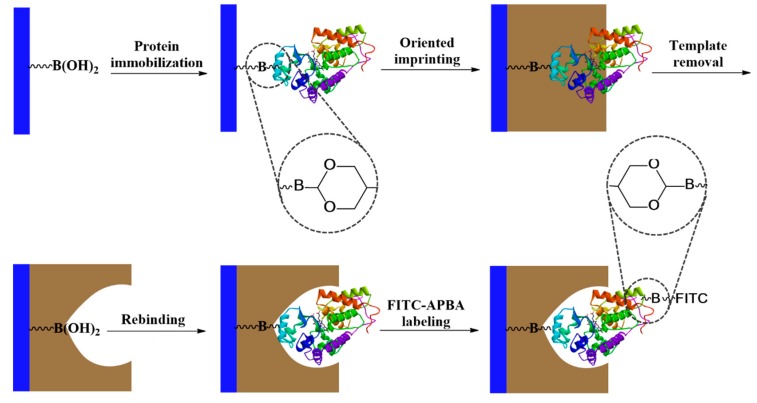
Schematic of glycoprotein imprinting using immobilized template, and detection of the molecularly imprinted polymer (MIP)-captured glycoprotein using boronic acid–fluorescein conjugate (BA–FITC) as a fluorescent probe.

The principle of glycoprotein detection used in this work is depicted in Scheme 1. The core–shell nanoparticles are composed of uniform silica coated with a thin layer of protein-imprinted polymer layer. Prior to the molecular imprinting reaction, the glycoprotein template was first immobilized on boronic acid–functionalized silica through a covalent boronate ester bond. After the template immobilization, dopamine polymerization was initiated by adding APS into the particles suspended in alkaline buffer. The reaction time was adjusted to control the thickness of the polymer layer to make sure that the imprinted sites were accessible for protein binding.

### 3.1. Characterization of Protein-Imprinted Core–Shell Nanoparticles

The protein-imprinted core–shell nanoparticles were characterized by dynamic light scattering, scanning electron microscopy (SEM), transition electron microscopy (TEM), thermogravimetric analysis, and elemental analysis. The experimental results were used to estimate the particle size and size distribution, the thickness of the imprinted polymer layer, and the content of the boronic acid in the composite materials.

#### 3.1.1. Characterization of Nanoparticles by DLS and Electron Microscopy

**Figure 1:**
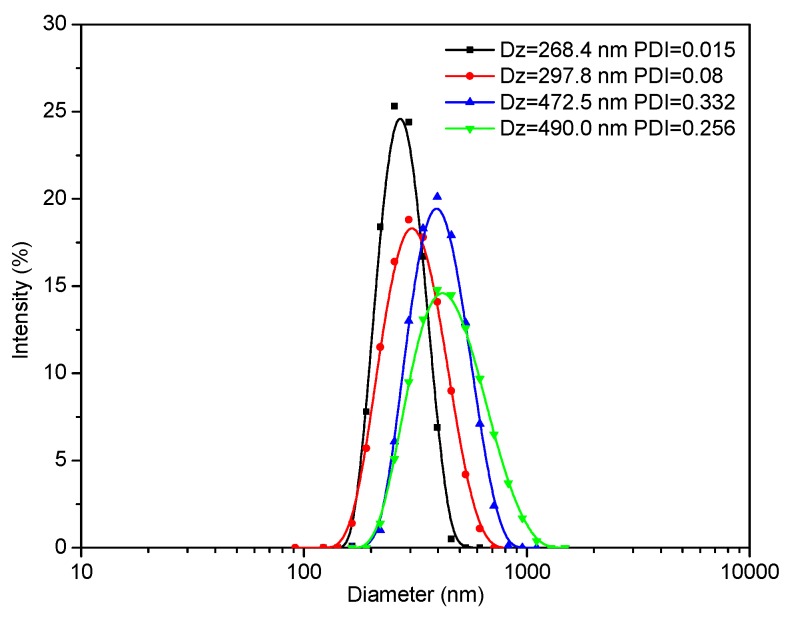
Hydrodynamic particle size of silica (**black**), silica@BA (**red**), horseradish peroxidase (HRP)-imprinted MIP (**blue**), non-imprinted polymer (NIP) (**green**) measured in water.

**Figure 2:**
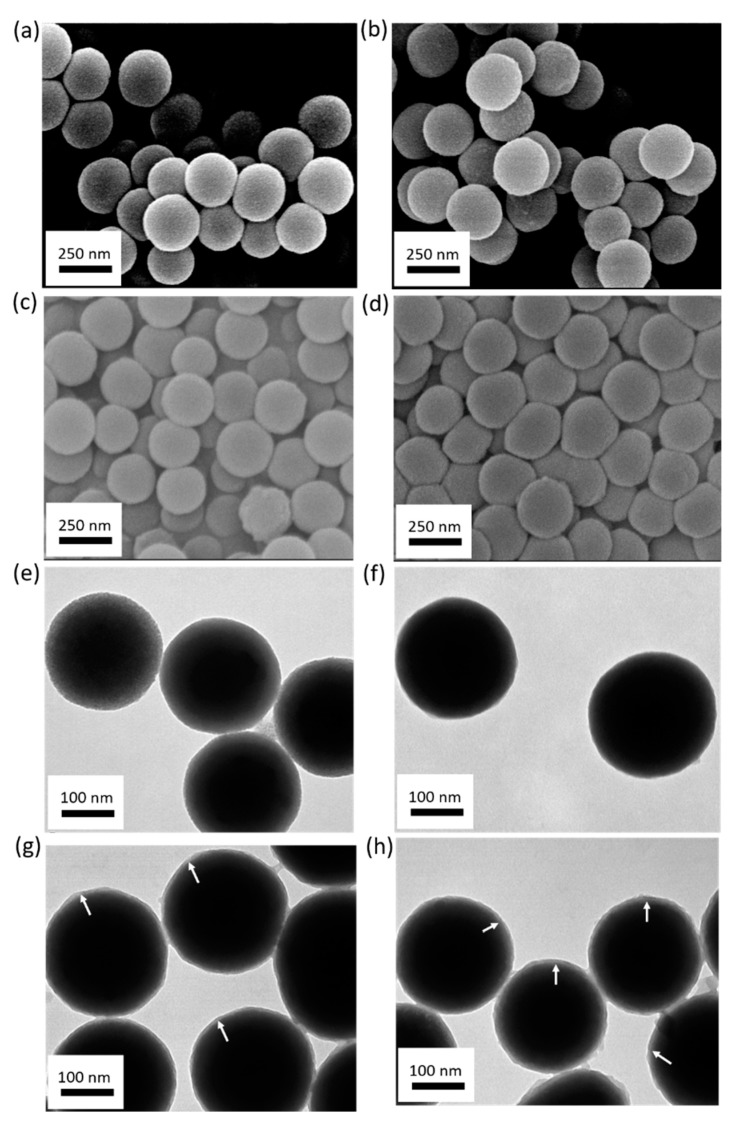
SEM images of (**a**) silica nanoparticles, (**b**) Si@BA, (**c**) HRP–MIP, and (**d**) NIP. TEM images of (**e**) silica nanoparticles, (**f**) Si@BA, (**g**) HRP–MIP, and (**h**) NIP.

The sizes of the different nanoparticles prepared were first measured by DLS. As shown in Figure 1, the hydrodynamic diameter of the silica particles was 268 nm, with a very narrow size distribution (Polydispersity index (PDI) = 0.015, black curve). After modification with the boronic acid, the hydrodynamic diameter of the particles increased to 298 nm (PDI = 0.08, red curve). The increased particle size was due to the modification of the silica core with APTES and the subsequent functionalization with the boronic acid. The hydrodynamic sizes of these particles appeared to be similar to those measured from the corresponding electron microscopy images (silica particles: Figure 2a,e; boronic acid-modified particles: Figure 2b,f).

After copolymerization of dopamine and APBA on the silica particle surface, a thin polymer layer (~6 nm) was observed on both the MIP and NIP particles (Figure 2g,h). The hydrodynamic diameters of the MIP and NIP particles were measured to be 470 nm and 490 nm, respectively (Figure 1, blue curve and green curve), which were larger than those determined by SEM (Figure 2c,d) and TEM (Figure 2g,h). The smaller particle size obtained by electron microscopy was due to the dehydration of the polymer layer in vacuum.

#### 3.1.2. Compositional Analysis of Nanoparticles 

**Figure 3:**
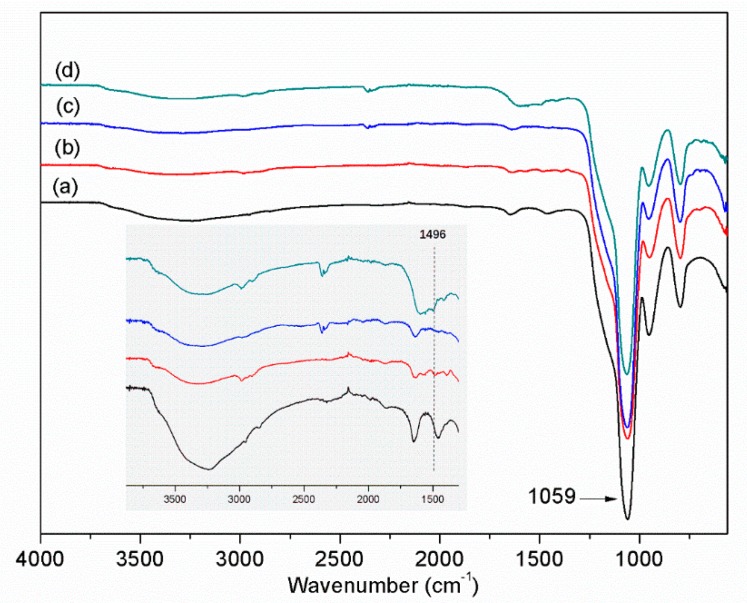
FT-IR spectra of (**a**) Si, (**b**) Si@NH_2_, (**c**) Si@BA, and (**d**) HRP–MIP.

The functional groups in the different nanoparticles were analyzed by FT-IR. The most distinct absorption peak at 1059 cm^−1^ is ascribed to the asymmetric vibration of Si−O−Si (Figure 3, curve a). The characteristic band at 1496 cm^−1^ is assigned to the amine groups from APTES (curve b). After the reaction with FPBA, the amine groups disappeared (curve c). Due to the weak IR signal of boronic acid, the signal of the boronic acid group was not observed. In the imprinted MIP particles, the signal of amine groups appeared again, which indicated the formation of polydopamine layer on the particle surface.

**Figure 4:**
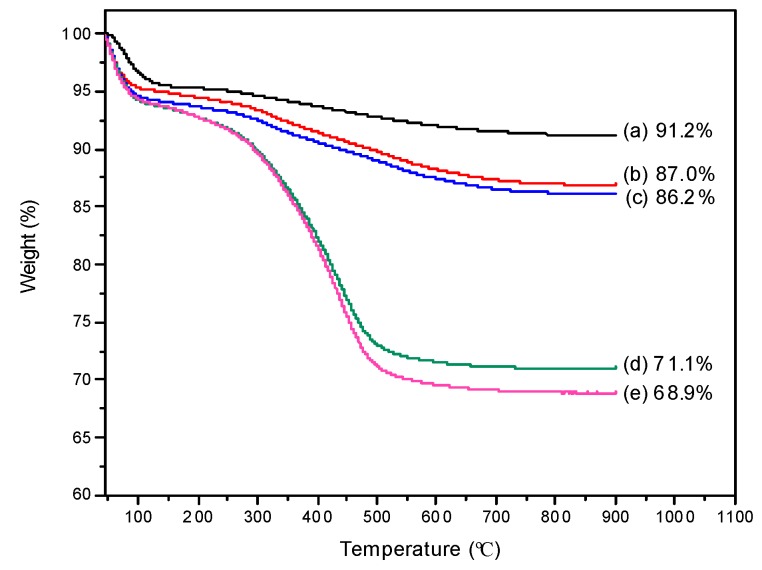
TGA analysis of (**a**) Si, (**b**) Si@NH_2_, (**c**) Si@BA, (**d**) HRP–MIP, and (**e**) NIP.

The content of organic materials in the different composite particles (Si@NH_2_, Si@BA, HRP–MIP, and NIP) was measured by thermogravimetric analysis (TGA) and differential thermogravimetric analysis (DTG). As shown in Figure 4 and Figure S1 (in supplementary material), there are three principle thermal transitions occurring in the temperature ranges of 45–250 °C, 250–350 °C, and 350–900 °C. The weight loss (~6%) below 250 °C can be attributed to the evaporation of residual organic solvent and water. The silica nanoparticles did not exhibit significant weight loss after 250 °C. The weight loss during the second thermal transition (250–350 °C) corresponds to the decomposition of APTES and BA molecules. The difference in organic content between Si and Si@NH_2_ (4.2%, Figure 4, curves a and b) was due to the modification of Si with ATPES. A further modification with BA resulted in a slightly increased organic content (0.8%) for Si@BA (Figure 4, curves b and c). Elemental analysis showed that the nitrogen and boron content in Si@BA particles were 0.52% and 0.3%, respectively. Therefore, the density of nitrogen-containing functional groups and boronic acid in Si@BA particle was calculated to be 0.37 mmol g^−1^ and 0.28 mmol g^−1^. Around 76% of the amino groups in Si@NH_2_ were converted into boronic acid groups in Si@BA. For the HRP–MIP and NIP particles, a large weight loss occurred when the temperature increased from 350 °C to 900 °C (Figure 4, curves d and e), due to the decomposition of the organic polymer layer.

### 3.2. Detection of Glycoprotein Captured by Imprinted Nanoparticles

In this work, we used three different glycoproteins (HRP, OVA, and TRF) as templates to prepare MIPs that have specific binding sites with a controlled orientation on nanoparticle surface. According to Scheme 1, part of the saccharides on the glycoprotein is used to immobilize the protein template on nanoparticle surface. After the imprinting polymerization and removal of the template, the imprinted site will be able to bind the target glycoprotein by forming covalent boronate ester bond with the same saccharide “epitope”. The principle of this “oriented molecular imprinting” has been demonstrated to be successful for preparing glycoprotein-binding MIPs in a number of previous studies [13,14,15,17,19,20,21]. Given that an oriented binding site for glycoprotein has been created, the next question is how to detect the target protein in a straightforward manner. We considered that a simple fluorescence measurement using a selective molecular probe is potentially useful for practical applications, because the measurement itself is fast and easy to perform. The important issue is to design a molecular probe that is able to bind the remaining surface epitopes of the captured glycoprotein, similar to the situation in the traditional sandwich assay for proteins.

**Figure 5:**
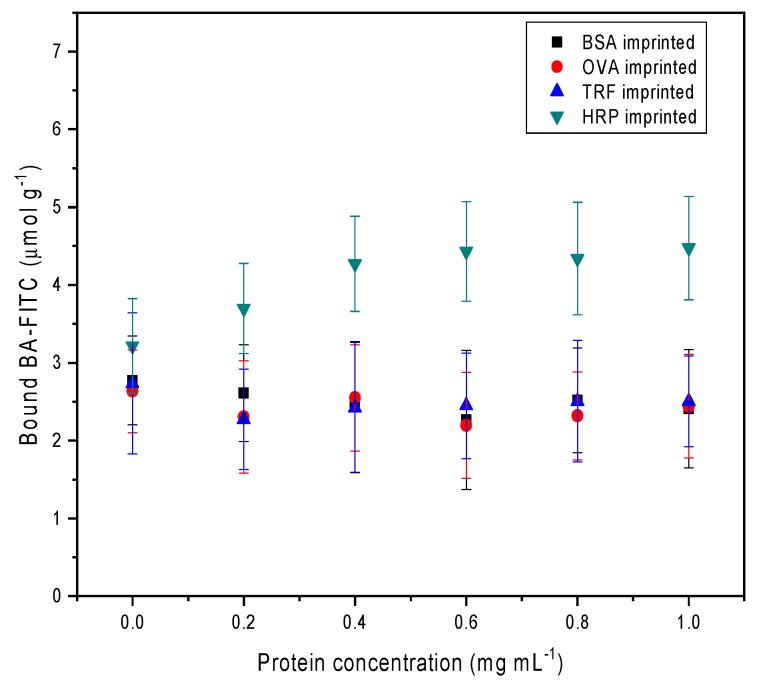
Detection of protein binding to imprinted particles using BA–FITC as a fluorescence probe. The MIP nanoparticles (3 mg) were first incubated with their corresponding protein templates (0.2–1.0 mg mL^−1^). After this step, BA–FITC was added, and the bound BA–FITC was measured using a fluorescence spectrometer.

To test the feasibility of the proposed sandwich assay, we used HRP, OVA, and TRF as model glycoproteins based on the following considerations: While HRP has multiple glycosylation sites [28] and is most likely to be captured by its MIP and subsequently detected by boronic acid–fluorophore conjugate, OVA and TRF only have a single glycosylation site [29,30] that will be masked after binding to their corresponding MIPs. Consequently, OVA and TRF captured by their corresponding MIPs will not respond to the boronic acid-modified fluorescence probe. Indeed, as shown in Figure 5, among the three glycoproteins, only HRP and its MIP were able to cause BA–FITC to bind to the solid particles in a dose-dependent fashion. The other two glycoproteins, OVA and TRF, and the nonglycosylated protein BSA, after being exposed to their corresponding MIPs, were unable to induce specific uptake of BA–FITC.

**Figure 6:**
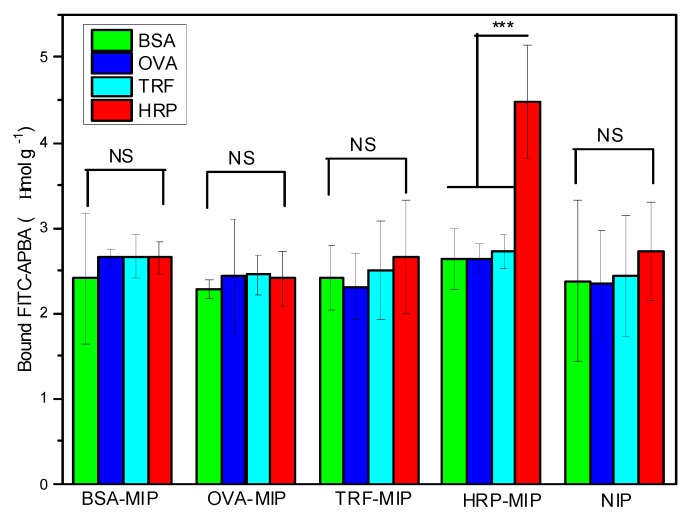
Selective detection of HRP captured by HRP–MIP using BA–FITC conjugate. The different imprinted nanoparticles (BSA–MIP, OVA–MIP, TRF–MIP and HRP–MIP) and NIP nanoparticles were first incubated separately with the four proteins before being exposed to BA–FITC. The concentration of the different proteins was fixed at 1.0 mg mL^−1^. *P* values were calculated by one-way analysis of variance (ANOVA) to estimate the statistically significant differences (*** *P* < 0.005). NS represented no significance. BSA: Bovine serum albumin, OVA: Ovalbumin, TRF: Transferrin.

To further verify that the enhanced BA–FITC binding in the HRP/HRP–MIP system was caused by the specific protein recognition from the imprinted polymer, we also measured the uptake of BA–FITC by the different composite particles that have been treated with the four proteins. As shown in Figure 6, it is only in the HRP/HRP–MIP system where specific uptake of the fluorescent probe could be observed. For all the other protein-MIP combinations, the binding of BA–FITC was much lower and was only caused by nonspecific adsorption, as that observed from the four protein–NIP mixtures (Figure 6, the last group of columns). To improve the imprinting effect and reduce the nonspecific adsorption, the protein imprinting process (polymerization reaction) will need to be further optimized.

## 4. Conclusions

In this work, we have demonstrated the possibility of replacing lectins/antibodies to develop simple sandwich assay for glycoproteins. For the first time, a small boronic acid-based fluorescent probe was combined with protein-imprinted nanoparticles to develop the sandwich assay. The present analytical setting is suitable to detect multiply glycosylated proteins (e.g., HRP) captured by their corresponding MIPs. For proteins with a single glycosylation site, other low molecular weight detection probes will need to be used, e.g., synthetic receptors that recognize a short peptide epitope located on the protein surface opposite to the carbohydrate. Our experimental results confirmed that well-constructed binding sites created by oriented protein imprinting can act as capture lectins/antibodies for the development of sandwich protein assays. The outstanding stability and low cost of the synthetic materials (MIPs and fluorescent probe) are particularly attractive for developing fast and simplified sandwich assays for not only glycoproteins, but also other biomacromolecular targets. For the present sandwich assay, one limitation is that the thickness of the imprinted polymer layer needs to be optimized to match the size of each macromolecular template. The nonspecific binding of the fluorescent probe also needs to be minimized by optimizing the binding condition.

## Supplementary Materials

The following are available online at http://www.mdpi.com/2073-4360/11/1/173/s1.
