# Supplementary material for: Towards Detection of Glycoproteins Using Molecularly Imprinted Nanoparticles and Boronic Acid-Modified Fluorescent Probe
Source: Polymers (Basel). 2019 Jan 18;11(1):173. doi: 10.3390/polym11010173 (PMC6401722; doi:10.3390/polym11010173)
Supplement: Supplementary file 1 [file polymers-11-00173-s001.pdf]

Supplementary Material

Towards detection of glycoproteins using molecularly imprinted nanoparticles and boronic acid-modified fluorescent probe

Lingdong Jiang [1], Rui Lu [1,2] and Lei Ye [1,*]

[1] Division of Pure and Applied Biochemistry, Department of Chemistry, Lund University, Sweden; lingdong.jiang@tbiokem.lth.se; lei.ye@tbiokem.lth.se

[2] Jiangsu Key Laboratory of Chemical Pollution Control and Resources Reuse, School of Environmental and Biological Engineering, Nanjing University of Science and Technology, China; rlu@njust.edu.cn

* Correspondence: lei.ye@tbiokem.lth.se; Tel.: +46-46-2229560 (L.Y.)

Results of DTG analysis

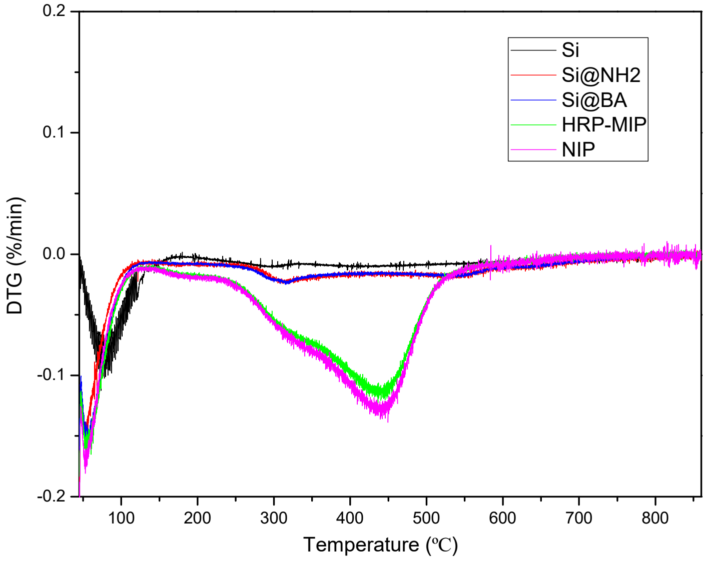

Figure S1. DTG analysis of Si (black), Si@NH_2 (red), Si@BA (blue), HRP-MIP (green) and NIP (purple).
